# Doing justice when incorporating sustainability into pre‐medical curricula

**DOI:** 10.1111/medu.15653

**Published:** 2025-03-01

**Authors:** Christian Moro, Michelle McLean, Charlotte Phelps

## 1 WHAT PROBLEMS WERE ADDRESSED?

There are increasing calls from students, universities and accrediting bodies to embed Planetary Health (including sustainability) in health professions education. With limited formal guidance, academics have been trialling different approaches. Crammed health professions programmes make it difficult to undertake curriculum changes. Major changes also require curriculum committee approval, which can take months, even years.

## 2 WHAT WAS TRIED?

As a stop‐gap measure that minimally increased student workload, we included a Planetary Health ‘fact’ on the bottom of a single relevant PowerPoint slide in each 2‐hour lecture across a 12‐week pre‐medical physiology subject, with references and links to further information relating to the health impacts of a changing climate. For example, the session introducing mucosal tissue highlighted the impact of pollution on the respiratory system. Other sessions discussed sports performance and outside temperature, disease outbreaks, or the environmental footprint of healthcare. To minimise any formal subject changes, the educator read the facts during lectures, but no time was devoted to discussion or assessment. The intention was to alert students to the environmental determinants of health and hopefully motivate them to follow up on the provided links. Towards the end of the 12‐week semester, students were surveyed to check whether this stop‐gap approach (the weekly fact) was sufficient to pique interest. Additionally, students were asked to define Planetary Health in an open‐ended question, with experts evaluating the definitions for accuracy.

## 3 WHAT LESSONS WERE LEARNED?

Only 30% (9 out of the 30 responders) of pre‐medical students reported following up on these facts. Despite this, there appeared to be some benefit in providing facts, as exemplified in this student comment: ‘My actions, behaviours or thoughts have not changed, but the facts have made me more conscious of the effects.’ From feedback analysis, the students' ability to provide a definition of Planetary Health, and their understanding of its core concepts, were, however, not enhanced by the sole provision of facts. To address this, the next iteration of the subject incorporated a formal introduction to Planetary Health, which included a five‐minute pre‐class video and a 10‐minute discussion in the first lecture.

Based on our experiences, we offer several recommendations for academics wanting to incorporate Planetary Health information within a subject. First, include a definition of Planetary Health at the outset, highlighting the interrelationship between the health of the planet and human health.^1^ Second, link the included content to the relevant Sustainable Development Goal (SDG). For example, by highlighting the impact of increasing global temperatures on human body performance (SDG13: Climate Action). Third, incorporate facts throughout the lectures, but refer to the Planetary Health definition and advance this concept each time a new fact is introduced. Fourth, encourage students to seek additional information on the environmental determinants of health and well‐being as they apply to the subject content. In conclusion, time is required to introduce Planetary Health in a subject (and preferably throughout the course). With careful planning, it is possible to integrate facts longitudinally across the semester without adversely impacting already crammed curricula as a stop‐gap approach while more wholesale curriculum planning is underway.

## AUTHOR CONTRIBUTIONS

**Christian Moro:** Conceptualization; data curation; formal analysis; investigation; methodology; project administration; validation; writing—review and editing; writing—original draft. **Michelle McLean:** Conceptualization; investigation; writing—original draft; methodology; validation; writing—review and editing; formal analysis; project administration; data curation. **Charlotte Phelps:** Data curation; formal analysis; project administration; methodology; validation; writing—review and editing; conceptualization; investigation; writing—original draft.

## CONFLICT OF INTEREST STATEMENT

All authors (C.M., M.M. and CP) declare no conflict of interest.

## ETHICS STATEMENT

Ethics was approved for the study by the Bond University Human Research Ethics Committee.

## Data Availability

The data that support the findings of this study are available from the corresponding author upon reasonable request.
